# Remote Health Monitoring in the Workplace for Early Detection of COVID-19 Cases during the COVID-19 Pandemic Using a Mobile Health Application: *COVIDApp*.

**DOI:** 10.3390/ijerph19010167

**Published:** 2021-12-24

**Authors:** Patricia Echeverría, Jordi Puig, José María Ruiz, Jordi Herms, Maria Sarquella, Bonaventura Clotet, Eugenia Negredo

**Affiliations:** 1 Infectious Diseases Department & Lluita Contra la Sida Foundation, Hospital Universitari Germans Trias i Pujol, 08916 Badalona, Spain; jpuig@flsida.org (J.P.); msarquella@flsida.org (M.S.); bclotet@irsicaixa.es (B.C.); enegredo@flsida.org (E.N.); 2 Doolehealth S.L. Digital Health Department, 08500 Barcelona, Spain; jmruiz@doolehealth.com (J.M.R.); jherms@doolehealth.com (J.H.); 3 AIDS Research Institute—IRSICAIXA, Hospital Universitari Germans Trias i Pujol, Universitat Autònoma de Barcelona, 08916 Badalona, Spain; 4 Infectious Diseases and Immunity, Centre for Health and Social Care Research (CESS), School of Medicine, University of Vic-Central University of Catalonia (UVic-UCC), 08500 Catalonia, Spain

**Keywords:** COVID-19, health application, workplace, early detection, remote health monitoring

## Abstract

*Background:* COVIDApp is a platform created for management of COVID-19 in the workplace. *Methods:* COVIDApp was designed and implemented for the follow-up of 253 workers from seven companies in Catalonia. The assessment was based on two actions: first, the early detection and management of close contacts and potential cases of COVID-19, and second, the rapid remote activation of protocols. The main objectives of this strategy were to minimize the risk of transmission of COVID-19 infection in the work area through a new real-time communication channel and to avoid unnecessary sick leave. The parameters reported daily by workers were close contact with COVID cases and signs and/or symptoms of COVID-19. *Results:* Data were recorded between 1 May and 30 November 2020. A total of 765 alerts were activated by 76 workers: 127 green alarms (16.6%), 301 orange alarms (39.3%), and 337 red alarms (44.1%). Of all the red alarms activated, 274 (81.3%) were activated for symptoms potentially associated with COVID-19, and 63 (18.7%) for reporting close contact with COVID-19 cases. Only eight workers (3.1%) presented symptoms associated with COVID-19 infection. All of these workers underwent RT-PCR tests, which yielded negative results for SARS-CoV2. Three workers were considered to have had a risk contact with COVID-19 cases; only 1 (0.4%) asymptomatic worker had a positive RT-PCR test result, requiring the activation of protocols, isolation, and contact tracing. *Conclusions:* COVIDApp contributes to the early detection and rapid activation of protocols in the workplace, thus limiting the risk of spreading the virus and reducing the economic impact caused by COVID-19 in the productive sector. The platform shows the progression of infection in real time and can help design new strategies.

## 1. Introduction

The disease caused by the SARS-CoV-2 virus, COVID-19, has become a global pandemic affecting around 200 countries worldwide, with more than 158.9 million people infected globally and more than 3.3 million related deaths as of 14 May 2021 [1,2]. The rapid spread of the infection and its severity in a considerable percentage of patients has necessitated unprecedented public health measures.

The impact of COVID-19 on the productive sector worldwide has been dramatic [3,4,5,6]. Spain is one of the countries most affected by the COVID-19 pandemic. Since the first confirmed case reported on 31 January 2020, there have been over 3.5 million people infected with COVID-19 and 79,281 related deaths [7]. The unprecedented economic and social impact of the pandemic [8,9] has affected all productive sectors of the economy, due to the high risk of transmission of COVID-19 in the workplace, which requires quick and innovative responses [6]. Companies should develop a model for their business activity that guarantees workers’ safety, reduces sick leave caused by COVID-19, and establishes risk control mechanisms to reduce or limit the impact of COVID-19 in the workplace.

In this sense, telemedicine has become a powerful tool that can help to mitigate the impact of the disease on healthcare and the use of healthcare resources. Thus, the implementation of a mobile application (app) whose main goal is the early detection of symptoms of COVID-19 can help to minimize transmission and thus prevent further infections in the productive sector. Such an app could also help to minimize unnecessary sick leave.

During the pandemic, the Spanish company Doole Health developed a unique tool (COVIDApp) to enable early detection of COVID-19 cases, self-isolation of suspected cases, and remote management of mild cases in the workplace through real-time monitoring of the worker.

COVIDApp was previously used during the first wave of the COVID-19 pandemic to provide assistance to nursing home staff and primary care clinicians involved in close monitoring of institutionalized persons and their contacts through remote medical care. COVIDApp thus opened a new channel for real-time communication [10].

The main objective of this manuscript is to describe the use of the telemedicine platform COVIDApp (as an innovative tool) to minimize the risk of transmission of COVID-19 infection in the work area through a new real-time communication.

We describe the application and design of the study, the results (which describe the monitoring of workers from different productive sectors), and finally the usefulness of the new strategy for the control of an epidemic are discussed, and compared with other similar applications.

## 2. Methods

### 2.1. Participants and Objectives

COVIDApp was implemented in seven companies in Catalonia for the follow-up of 253 workers from different productive sectors of the economy (food and drink, real estate, and tourism).

The manuscript only contains data obtained through the use of existing information in a database that does not contain personal data; therefore, ethical approval is not required.

The data reported in this manuscript were registered on the platform between 1 May 2020 and 30 November 2020.

COVIDApp aims to ensure workplace safety through the following: (1) rapid detection of people with symptoms of COVID-19; (2) rapid detection of persons who have been in close contact with a COVID-19 case, and (3) remote activation of protocols such as referral to a primary care center for reverse transcription–polymerase chain reaction (RT-PCR) assay and isolation of suspected cases.

### 2.2. COVIDApp Use and Dynamics

The app was installed on the smartphones of all workers involved in the study, and access was provided through a username and primary password.

Electronic informed consent was obtained from all participants who accepted the clauses, authorized the treatment, and transferred the data (that was collected daily by voluntary introduction on the platform) to be used for scientific purposes by the person responsible for the treatment. These data were anonymized and dissociated in compliance with the GDPR (General Data Protection Regulation).

**Figure 1:**
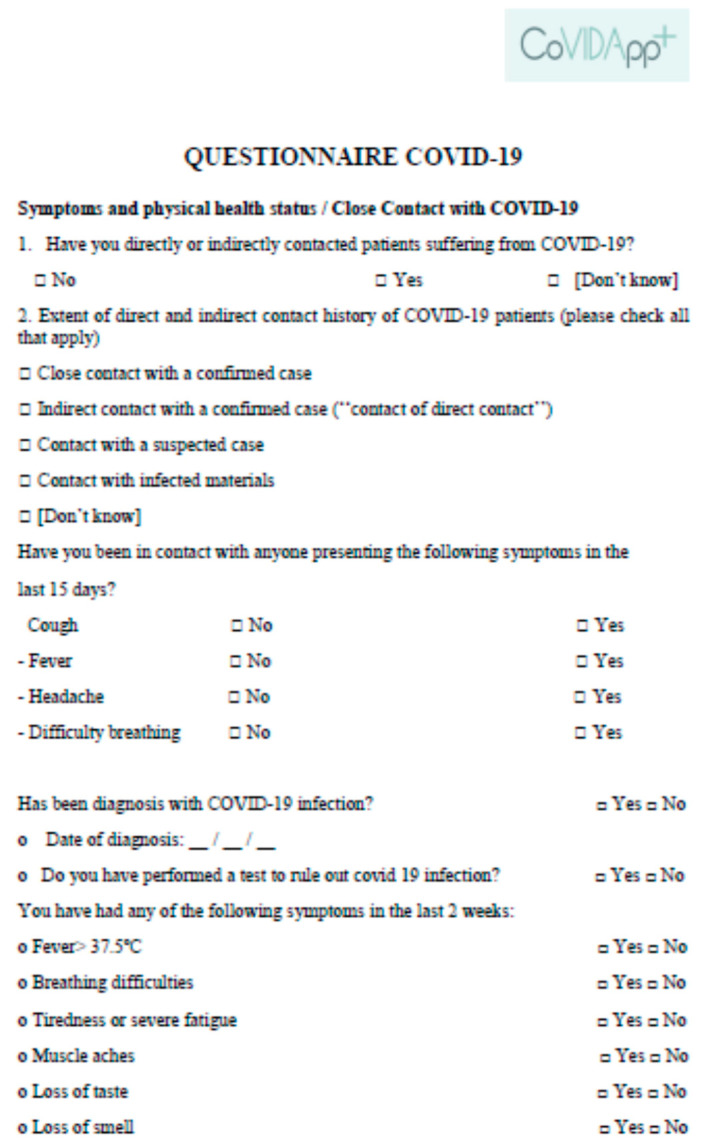

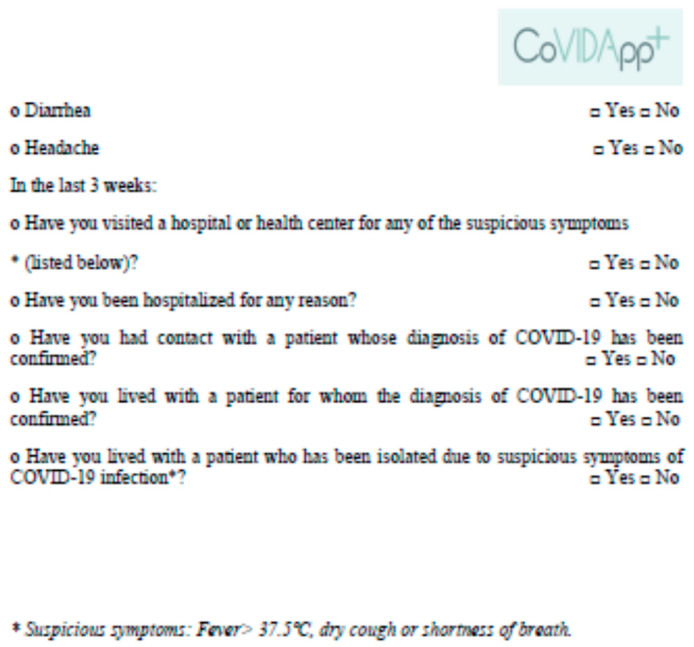
Questionnaire filled out daily by the workers to report symptoms or close contact with COVID-19.

COVIDApp collected information about the workers in real time, namely, symptoms related to COVID-19 according to the World Health Organization (WHO) [11] (e.g., cough, headache, dyspnea, vomiting, diarrhea, fever) and close contact with COVID-19 cases through a daily questionnaire filled out before coming to work. All the required information was collected in a questionnaire designed with a basic language (easily understandable) and only required simple answers “yes” or “no”. Workers received an automatic message sent by the app as a reminder to fill out the questionnaire (Figure 1).

A specific computer program was created to check all the information provided by the worker in real time and to ensure that healthcare personnel could provide additional clinical information, monitor worker data, and answer messages. The application did not save any data. Data were obtained for viewing only by prior request to the server and encrypted using Secure Hash Algorithm 1 with the 256-bit Secure Sockets Layer security certificate.

An immediate alert in the form of color alarms was sent to the healthcare team via the app when a worker reported close contact or signs or symptoms related to COVID-19: green alarms were activated when the worker reported symptoms unrelated to COVID-19 infection (low suspicion); orange alarms were activated when the worker reported symptoms that could be associated with COVID-19 infection (e.g., myalgia, headache) (medium suspicion); and red alarms were activated when the worker reported symptoms more likely to be associated with COVID-19 infection (e.g., dyspnea, fever) or possible contact with COVID cases (high suspicion). Three days with orange symptoms triggered a red alarm, as did the combination of fever, cough, fatigue, diarrhea, and/or anosmia.

Following the alarm, a clinical assessment by the healthcare team was planned within 2 h, and epidemiological measures were recommended, such as self-isolation of suspected cases and contacts and performing RT-PCR testing in the following 24 h. Suspected cases were isolated until the RT-PCR result was available (within 24 h), and those whose test was positive for SARS-CoV2 remained in quarantine and were monitored through a primary care center.

Finally, mild cases received support through COVIDApp from the healthcare team and primary care centers (all remained isolated at home), while severe cases were transferred to the hospital.

The application provided a daily report of the number of suspected or confirmed COVID-19 cases, isolated cases, and workers remaining asymptomatic in the workplace. These reports were given to the heads of each company to show changes in the situation and interventions.

## 3. Results

Only 55% of the workers answered the symptom questionnaire daily during the first month of follow-up. However, during the second month, the number of reports increased to 86%, reaching 100% from the fourth month onward (coinciding with the second wave of COVID-19 in Spain).

**Figure 2:**
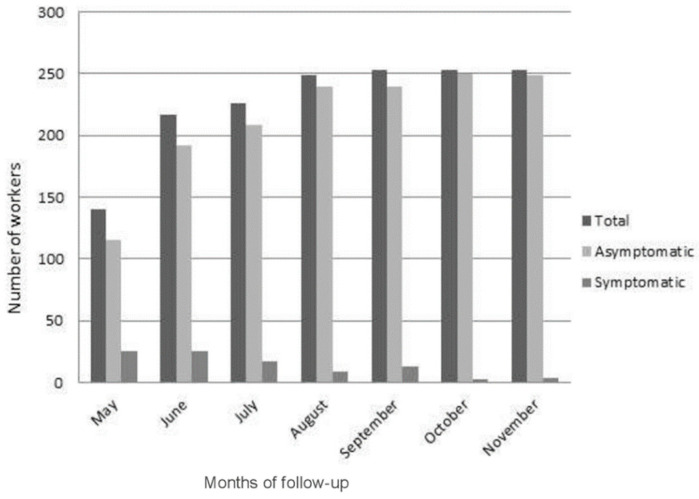
Number of workers who completed the questionnaire (total), number of these workers who reported signs and/or symptoms related to COVID-19 (symptomatic), and number of workers remaining as symptomatic between 1 May 2020 and 30 November 2020.

During the 7 months of follow-up with COVIDApp, slightly more 90% of the workers remained asymptomatic; 9.8% experience symptoms at the beginning. The number of symptomatic workers decreased progressively to 1.6% at the end of November (Figure 2).

**Figure 3:**
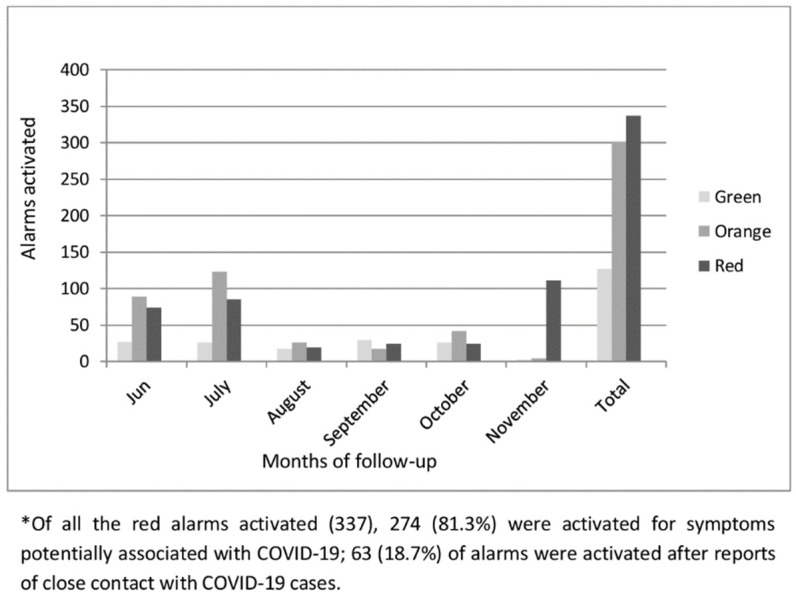
Total number of alerts sent by workers to the COVIDApp healthcare team between 1 May 2020 and 30 November 2020.

A total of 765 alerts were activated by 76 workers: 127 green alarms (16.6%) by 22 workers; 301 orange alarms (39.3%) by 52 workers; and 337 red alarms (44.1%) activated by 58 workers (Figure 3). 

Of all the red alarms activated, 274 (81.3%) were activated because of symptoms potentially associated with COVID-19 by 43 workers, and 63 (18.7%) were activated by 10 workers owing to close contact with a COVID-19 case.

**Figure 4:**
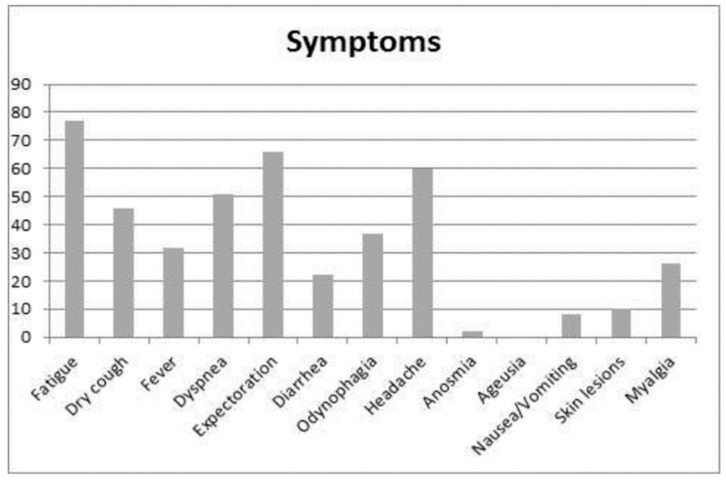
Symptoms most frequently reported by workers.

The most common symptoms were fatigue (17.6%), expectoration (15.1%), headache (13.7%), dyspnea (11.6%), and dry cough (10.5%) (Figure 4).

After a telephone interview with healthcare personnel, only 8 of 43 workers (18.6%) with symptoms potentially associated with COVID-19 presented symptoms really suggestive of COVID-19 infection. These workers underwent RT-PCR tests, which were negative for SARS-CoV2 in all cases.

Of the 63 (18.7%) red alarms activated by 10 workers due to a close contact with a COVID-19 case, only three workers (30%) were considered to have had close contact with COVID-19 cases. Of these, only one asymptomatic worker (10%) had a positive RT-PCR result, requiring the activation of protocols, isolation at home, and study of contacts in the work environment. The contact study reported that the COVID-19 case had been in close contact with only three workers, all of whom required isolation. Assessment with RT-PCR yielded negative results for SARS-CoV2 in all cases.

No severe cases of COVID-19 were reported.

## 4. Discussion

COVIDApp was created to assist in the management of confirmed or suspected cases of COVID-19. The platform was tested in real clinical settings [10,12]. In the present study, COVIDApp was successfully implemented for the follow-up of 253 workers belonging to different productive sectors in Catalonia. Assessment was based on two actions: first, the early detection and management of close contacts and potential cases of COVID-19; and second, the rapid remote activation of protocols. The main objectives of this strategy were to minimize the risk of transmission of COVID-19 infection in the work area through a new real-time communication channel and to avoid unnecessary sick leave.

Many applications have been designed worldwide to transform contact tracing of SARS-CoV-2 outbreaks. However, uptake has been low [13,14]. Most of the many digital collection tools being developed for COVID-19 have been configured to offer a single assessment of symptoms and thus provide semi-personalized recommendations for further evaluation [15,16].

With data and guidelines still in development, support tools are needed to manage the situation owing to the complete saturation of the national health system. In this context, telemedicine could be promoted for early diagnosis, patient isolation, and contact tracing. Many telemedicine platforms are being developed focused on the area of telemedicine and telecare. However, different to our application, no epidemiological data have been yet published from these platforms [17,18]. Preliminary data on telemedicine, particularly video consultations, have been promoted and scaled up to reduce the risk of transmission using this information for the monitoring of symptomatic individuals in the United Kingdom [19] and the United States of America. [20,21]. In China, a telemedicine platform was designed including a COVID-19 informational page, which updated the latest information in real time, with instructions for quarantine processes at home, personal protection applications, and time for seeking medical attention. The telemedicine platform also included an online consulting clinic, where experts were available 24 h/day. Experts could conduct preliminary screenings through remote consultation, which avoided the risk of cross infection in the hospitals and relieved pressure away from designated hospitals. This platform was similar to ours, and, like ours, showed a decrease in latent COVID-19 infection [22].

In France, Clément Cormi et al. [23] has described the use of telemedicine to advise and support elderly people in nursing homes through a website that enables direct contact between a senior geriatrician and centers for elderly people. Much like our system, this approach enables diagnosis and monitoring cases with COVID-19 in a care setting by mobile teams.

COVIDApp was rapidly accepted and used by the workers, probably because they knew the app was supported by a medical team that responded quickly. The fact that the questionnaire had to be completed daily, with updated information on the measures to be taken, reiterated the importance of implementation of these measures. In addition, the heads of the companies also reported their confidence in COVIDApp, since the app helped them to manage a situation without precedent.

In our study, an increasing percentage of the workers remained asymptomatic during the pandemic, thus confirming the effectiveness of preventive measures in the workplace. In addition, workers were fully aware of the steps to follow if they presented symptoms compatible with COVID-19 or had come into contact with the virus [24,25,26]. Monitoring of close contacts plays a large role in COVID-19 infection, and early detection (reported through COVIDApp in real time) enabled us to reduce the risk of contacts in the workplace and thus reduce the number of persons requiring sick leave due to COVID-19.

During the 7 months of follow-up, many alerts (765) were sent to the COVIDApp healthcare team through activation of an alarm system, thus indicating the clear concern of users during these months. However, only 18.6% of workers presented symptoms potentially related to COVID-19 infection, and three workers had close contact with COVID-19 cases; in other words, COVIDApp made it possible to detect cases early and implement safety measures.

All workers with suspicion of COVID-19 infection underwent RT-PCR. Only one case of COVID-19 (0.4%) was confirmed. This very low incidence is probably related to the daily reporting of contacts and symptoms via the application, the reinforcement of hygiene measures, and the confidence inspired by the feedback and constant advice from the healthcare team.

In addition to the early detection of COVID-19 cases, COVIDApp facilitated stratification of the risk of exposure and immediate decision making, thus decreasing the number of unjustified isolations within the company. This action, in turn, decreased the number of requests for sick leave due to COVID-19 and, therefore, costs and disruption.

Previous studies have demonstrated that more intensive implementation of workplace a measure responding to COVID-9 reduces employees’ psychological distress and enables them to maintain their work performance [27,28,29,30].

We consider that the current study has two major limitations: first, that general characteristic of participants is not available since these data were not collected on the platform, and second, data were self-reported. However, the health team called all workers with probable symptoms or close contact and asked questions to identify those with more risk.

While our strategy was based on detection and monitoring of suspected cases, it also had a double epidemiological objective, namely, to reduce transmission in the workplace and to monitor symptoms reported by workers.

## 5. Conclusions

In summary, COVIDApp is an innovative tool that could help to rapidly detect and remotely monitor suspected and confirmed cases of COVID-19 in public or private companies, thus limiting the risk of spreading the virus, as well as unjustified isolations. In addition, the platform shows the characteristics and progression of the situation in real time, thus facilitating the design of strategies tailored to a specific setting.

## Data Availability

Data is contained within the article.
